# Clinical assessment of a novel machine‐learning automated contouring tool for radiotherapy planning

**DOI:** 10.1002/acm2.13949

**Published:** 2023-03-04

**Authors:** Yunfei Hu, Huong Nguyen, Claire Smith, Tom Chen, Mikel Byrne, Ben Archibald‐Heeren, James Rijken, Trent Aland

**Affiliations:** ^1^ Icon Cancer Centre Concord Rusty Priest Building Concord Repatriation Hospital Concord NSW Australia; ^2^ ICON Core Office South Brisbane QLD Australia; ^3^ Icon Cancer Centre Springfield Cancer Care Centre Mater Private Hospital 30 Health Care Dr Springfield Queensland Australia; ^4^ Icon Cancer Centre Wahroonga Sydney Adventist Hospital Sydney Australia; ^5^ Icon Cancer Centre Windsor Gardens Windsor Gardens South Australia Australia

**Keywords:** automated contouring, efficiency and quality, machine learning, radiotherapy planning

## Abstract

Contouring has become an increasingly important aspect of radiotherapy due to inverse planning. Several studies have suggested that the clinical implementation of automated contouring tools can reduce inter‐observer variation while increasing contouring efficiency, thereby improving the quality of radiotherapy treatment and reducing the time between simulation and treatment. In this study, a novel, commercial automated contouring tool based on machine learning, the AI‐Rad Companion Organs RT™ (AI‐Rad) software (Version VA31) (Siemens Healthineers, Munich, Germany), was assessed against both manually delineated contours and another commercially available automated contouring software, Varian Smart Segmentation™ (SS) (Version 16.0) (Varian, Palo Alto, CA, United States). The quality of contours generated by AI‐Rad in Head and Neck (H&N), Thorax, Breast, Male Pelvis (Pelvis_M), and Female Pelvis (Pevis_F) anatomical areas was evaluated both quantitatively and qualitatively using several metrics. A timing analysis was subsequently performed to explore potential time savings achieved by AI‐Rad. Results showed that most automated contours generated by AI‐Rad were not only clinically acceptable and required minimal editing, but also superior in quality to contours generated by SS in multiple structures. In addition, timing analysis favored AI‐Rad over manual contouring, indicating the largest time saving (753s per patient) in the Thorax area. AI‐Rad was concluded to be a promising automated contouring solution that generated clinically acceptable contours and achieved time savings, thereby greatly benefiting the radiotherapy process.

## 1 INTRODUCTION

It has been estimated that approximately half of all cancer patients will benefit from radiotherapy treatment. Meanwhile, the burden on radiotherapy infrastructure is anticipated to increase in line with the incidence of cancer.^1^ To fully exploit the advantages of inverse planning in radiotherapy, all target volumes and surrounding organs at risk (OARs) must be contoured before treatment planning. This process may be repeated multiple times during a treatment course because of tumor response or changes in patient weight or anatomy. When manual contouring is performed, large inter‐observer variations have been reported^2^, ^3^, ^4^ and are considered one of the largest sources of uncertainty in radiotherapy.^5^ For some tumor locations, inconsistencies in target volume definition may dominate all other errors in planning and delivery.^6^ Although standardized guidelines and anatomy atlases have addressed the issue to a certain extent, automated segmentation using artificial intelligence (AI) has potential in both minimizing inter‐observer variations^7^ and significantly reducing contouring time while improving planning efficiency.^8^

As an emerging field of computer science, AI attempts to emulate human‐like intelligence by using computer software and algorithms to perform specific tasks without direct human input.^9^, ^10^, ^11^ One of the subcategories of AI, machine learning (ML), uses computer software, modelling, and algorithms to detect patterns and correlation through the learning process by providing it with databases of raw data.^9^ While ML has several applications in radiotherapy,^12^, ^13^, ^14^ its use in contouring has drawn substantial interest owing to the potential benefit to efficiency and consistency.^15^, ^16^, ^17^, ^18^ The ML approach is to learn the structure labelling of each image voxel directly, more flexibly incorporating prior knowledge in the form of parameterized models. Successful techniques include the use of statistical and decision‐learning classifiers,^19^, ^20^ and, more recently, deep learning.^21^, ^22^, ^23^

A new automated segmentation tool, the AI‐Rad Companion Organs RT™ (AI‐Rad) contouring software (Version VA31) (Siemens Healthineers, Munich, Germany), has been introduced in the authors’ department. The software was based on the method proposed by Ghesu et al.^24^ that reformulated the detection problem as a behavior learning task for an artificial agent. In other words, an artificial agent is trained not only to distinguish the target anatomical object from the rest of the body, but also to find the object by learning and following an optimal navigation path to the target object in the imaged volumetric space. The purpose of this study was to evaluate the quality of contours generated by AI‐Rad in different anatomical areas and, where possible, compare it to the quality of contours generated by Smart Segmentation™ (SS) (Version 16.0) (Varian, Palo Alto, CA, USA), a commercial automated segmentation solution that has been verified by various studies^25^, ^26^ and implemented clinically at the authors’ department. A timing analysis was also performed to explore potential time savings achieved by AI‐Rad compared to manual delineation.

## 2 METHODS

### 2.1 Clinical data collection

A total of 28 patients, composed of eight H&N patients, five thorax patients, five breast patients, five male pelvis patients, and five female pelvis patients, were retrospectively selected from patients treated at the authors’ department. The planning CT datasets of these patients were subsequently reviewed, during which it was noted that the collected patients varied in terms of body mass index (BMI), geometry (e.g., disease side, arm position and presence of breast implants in breast patients), and setup position (e.g., use of the Standard Wing Board™ vs. the Type S™ Overlay Board in breast patients). In addition, all CT datasets were free of significant artefacts and acquired in the head‐first supine (HFS) position.

### 2.2 Manual contouring

A radiation therapist (RTT) with at least 10 years of experience in radiotherapy contoured the major OARs that were commonly required for radiotherapy planning on the CT datasets of the 28 patients. The RTT performed the contouring task in accordance with clinical practice at the institution. Previous studies have demonstrated comparable contouring accuracy between RTTs and radiation oncologists (ROs).^27^, ^28^ Datasets were contoured in accordance with the Radiation Therapy Oncology Group (RTOG) contouring consensus.^29^ The time required to contour each OAR was recorded by the RTT.

### 2.3 AI‐Rad automated contouring

The AI‐Rad software provides a cloud‐based solution to automated contouring. To run an automated contouring session, a CT dataset is first uploaded to the cloud software from either the CT scanner or the treatment planning system (TPS). The system then determines the applicable template of structures using a user‐defined DICOM tag, which, in this study, was (0018, 0015) (Body Part Examined) before automated contours are generated. If no user‐defined templates are available, then all structures that can be automatically generated by the software and are present on the CT dataset are contoured by the system. The completed structure set is automatically transferred back to the TPS. During the entire process, the only step that requires user interaction is to upload the CT dataset.

In this study, once the structure set generated by AI‐Rad was imported into the TPS, it was reviewed and, if necessary, adjusted by the same RTT who did the manual contouring task. The time of this process was recorded as the time required for AI‐Rad to generate automated contours. The time required by the AI‐Rad software to process the CT dataset and generate contours was not included in the above timing data, as it significantly depends on system configurations. This study observed that under the trial setup (analogous to the clinical setup), it took less than 30 s for the AI‐Rad software to generate the required structures and import them to the TPS, or less than 5 s for each structure. Therefore, the impact of not including this process in the timing analysis was small. However, it should be noted that this time depends on system configuration, and whether the processing speed will be compromised upon large batches of patients requires further investigation.

During the review process, the RTT scored the “Degree of Editing,” a commonly used subjective evaluation metric,^30^, ^31^ of each automated contour according to the criteria listed in Table 1.

**TABLE 1:** Evaluation criteria used to assess the “Degree of Editing” of each automated contour.

**No edits required**	No changes made to the structure
**Minor edits required**	Less than 10% of slices requiring small edits
**Moderate edits required**	More than 10% of slices requiring small edits, or large edits required to a small number (10%) of slices
**Major edits required**	Edits not described in the above categories, up to and including the deletion and recontouring of the structure
**Not applicable**	Not relevant to the fraction or not assessed

### 2.4 SS automated contouring

The same structures were automatically contoured by SS, an atlas‐based automated contouring solution. Three SS atlases, each composed of 30–40 patients, were previously created from the datasets of retrospective clinical patients and clinically validated at the authors’ department, namely a H&N atlas, a breast atlas, and a pelvis atlas.^32^ In this study, the eight H&N patients were contoured by the H&N atlas, the five breast and five lung patients by the breast atlas, and the five female and five male pelvis patients by the pelvis atlas. Some OARs generated by AI‐Rad did not have a corresponding model in the SS. For these structures, no comparison was performed between AI‐Rad and SS.

### 2.5 List of contours

Table 2 lists contours generated manually, by AI‐Rad, and by SS.

**TABLE 2:** Contours generated manually, by AI‐Rad, and by SS.

Structure	No. of patients (n)	Manual	AI‐Rad	SS
A_Aorta	5	Y	Y	N
Bladder	10	Y	Y	Y
Brain	8	Y	Y	Y
Brainstem	8	Y	Y	Y
BreastL	5	Y	Y	Y
BreastR	5	Y	Y	Y
Esophagus	5	Y	Y	N
Femur_L	10	Y	Y	Y
Femur_R	10	Y	Y	Y
Globe_L	8	Y	Y	Y
Globe_R	8	Y	Y	Y
Heart	10	Y	Y	Y
Larynx	8	Y	Y	N
Lens_L	8	Y	Y	N
Lens_R	8	Y	Y	N
Lung_L	10	Y	Y	Y
Lung_R	10	Y	Y	Y
Mandible	8	Y	Y	N
OpticNrv_L	8	Y	Y	Y
OpticNrv_R	8	Y	Y	Y
Oral Cavity	8	Y	Y	N
Parotid_L	8	Y	Y	Y
Parotid_R	8	Y	Y	Y
Prostate	5	Y	Y	Y
Rectum	10	Y	Y	Y
SeminalVesicle	5	Y	Y	Y
SpinalCanal	10	Y	Y	Y
SpinalCord	8	Y	Y	Y

### 2.6 Comparison of contouring quality

Several quantitative metrics were calculated to assess the quality of automated contours, namely Dice similarity coefficient (DSC), sensitivity, precision, and Hausdorff distance (HD), with the structures manually contoured by the RTT as the ground truth.

DSC between respective volumes, A and B, is defined as:

(1) $$\mathrm{DSC}=2\times \mathrm{volume}\left(A\cap B\right)/\left(\mathrm{volume}\left(A\right)+\mathrm{volume}\left(B\right)\right)$$

DSC approaches 1.0 when two structures overlap exactly. One study has recommended a DSC of 0.7 to be considered a good overlap,^33^ whereas others have instead suggested 0.8.^34^, ^35^ DSC can provide false impression of high agreement.^35^ The metric can over‐penalize small structures whilst exaggerating the agreement of large structures.^36^ Therefore, three other quantitative metrics, that is, sensitivity, precision, and HD, are introduced.

Sensitivity is defined as:

(2) $$\mathrm{Sensitivity}=\mathrm{volume}\left(A\cap B\right)/\mathrm{volume}\left(B\right)$$

Sensitivity is the fraction of true positives divided by all actual positive cases in a population. If contour A is taken to be analogous to positive results and contour B to be positive cases, then sensitivity is the intersection of the two, divided by B. Sensitivity approaches 1.0 when all parts of the “true” contour is included in the automated contour.

Precision is defined as:

(3) $$\mathrm{Precision}=\mathrm{volume}\left(A\cap B\right)/\mathrm{volume}\left(A\right)$$

Precision refers to the number of true positive results divided by the sum of true positives and false positives, and therefore is the intersection of A and B divided by A. Precision approaches 1.0 when the automated contour only includes parts of the “true” contour.

HD is defined as the maximum Euclidean distance from each point in the ground‐truth contour to the nearest point in the automated contour.^37^ It has a minimum value of 0, indicating perfect agreement, and no maximum value. In this study, a sampling value of 20 was used to speed up the calculation. This works by only considering 1 in every 20 points on the ground‐truth contour, but still every point on the automated contour. It is approximated that a sample rate of 20 would introduce an average error of 3% for the HD.

The above metrics were calculated using an in‐house C# application implemented in the TPS.

### 2.7 Timing analysis

The automated contouring time of AI‐Rad was calculated as the time required by the RTT to review and adjust the specific structure to clinical standards. This time was subsequently compared to the time spent by the RTT to manually contour the same structure.

### 2.8 Statistical analysis

Quantitative data were expressed as Mean ± Standard Deviation (SD). The Jarque‐Bara test was used to assess the sample normality of DSC and HD. For data that were normally distributed, pairwise comparison was conducted with the Student's independent *t*‐test. The significance level was defined at *p* < 0.05.

## 3 RESULTS

Table 3 shows the DSCs of different automated contours delineated by AI‐Rad and SS against the manual contours. The last two columns of the table list the *p*‐values of the pairwise comparison, and the superior method if the difference is statistically significant (*p* < 0.05).

**TABLE 3:** DSCs of different structures between AI‐Rad and manual contours and SS and manual contours.

		AI‐Rad vs. manual		SS vs. manual			
Structure	No. of cases (*n*)	Mean	SD	Mean	SD	*p*‐Value	Superior method
A_Aorta	5	0.843	0.050				
Bladder	10	0.896	0.078	0.836	0.105	0.037	AI‐Rad
Brain	8	0.972	0.002	0.970	0.002	0.018	AI‐Rad
Brainstem	8	0.818	0.041	0.843	0.031	0.090	
BreastL	5	0.889	0.027	0.839	0.050	0.014	AI‐Rad
BreastR	5	0.892	0.041	0.854	0.031	0.045	AI‐Rad
Esophagus	5	0.747	0.056				
Femur_L^a^	10	0.934	0.027	0.897	0.091		
Femur_R^a^	10	0.939	0.017	0.897	0.094		
Globe_L	8	0.893	0.034	0.798	0.043	0.001	AI‐Rad
Globe_R	8	0.889	0.040	0.755	0.053	3.9E‐05	AI‐Rad
Heart^a^	10	0.931	0.016	0.893	0.036	0.008	
Larynx	8	0.405	0.037				
Lens_L	8	0.717	0.081				
Lens_R	8	0.722	0.060				
Lung_L^a^	10	0.958	0.021	0.967	0.012	0.201**^a^**	
Lung_R	10	0.965	0.014	0.964	0.016	0.473	
Mandible	8	0.851	0.031				
OpticNrv_L	8	0.531	0.104	0.209	0.243	0.010	AI‐Rad
OpticNrv_R	8	0.468	0.119	0.260	0.170	0.033	AI‐Rad
Oral Cavity	8	0.824	0.025				
Parotid_L	8	0.742	0.078	0.705	0.059	0.112	
Parotid_R	8	0.763	0.046	0.749	0.056	0.433	
Prostate	5	0.839	0.063	0.761	0.187	0.105	
Rectum	10	0.722	0.115	0.517	0.207	0.007	AI‐Rad
SeminalVesicle	5	0.652	0.080	0.594	0.247	0.232	
SpinalCanal	10	0.691	0.062	0.671	0.003	0.705	
SpinalCord	8	0.787	0.044	0.711	0.071	0.032	AI‐Rad

**^a^** Data were not normally distributed, and therefore Student's *t*‐test was not performed.

Table 4 shows the HDs of different automated contours delineated by AI‐Rad and SS against the manual contours. The last two columns of the table list the *p*‐values of the pairwise comparison, and the superior method if the difference is statistically significant (*p* < 0.05).

**TABLE 4:** HDs of different structures between AI‐Rad and manual contours and SS and manual contours.

		AI‐Rad vs. manual		SS vs. manual			
Structure	No. of cases (n)	Mean	SD	Mean	SD	*p*‐Value	Superior method
A_Aorta	5	15.6	10.7				
Bladder	10	9.7	6.8	12.2	5.0	0.060	
Brain	8	18.5	11.2	18.3	9.4	0.866	
Brainstem	8	8.9	2.5	5.5	0.8	0.005	SS
BreastL	5	25.0	9.2	35.5	14.0	0.101	
BreastR	5	21.6	5.6	27.6	9.8	0.047	AI‐Rad
Esophagus	5	15.9	7.6				
Femur_L	10	6.2	4.1	8.3	3.7	0.236	
Femur_R^a^	10	5.5	2.5	8.5	5.0		
Globe_L	8	2.7	0.6	4.8	1.0	0.002	AI‐Rad
Globe_R	8	2.8	1.0	5.0	1.0	0.002	AI‐Rad
Heart^a^	10	11.1	3.9	16.4	8.8		
Larynx	8	15.2	2.3				
Lens_L^a^	8	1.6	1.8				
Lens_R	8	1.7	0.5				
Lung_L	10	21.7	7.1	13.7	8.0	0.016	SS
Lung_R	10	28.3	12.1	16.7	14.5	0.045	SS
Mandible^a^	8	14.8	4.9				
OpticNrv_L^a^	8	4.1	3.9	19.5	5.1		
OpticNrv_R	8	3.7	2.4	17.0	3.6	2.8E‐05	AI‐Rad
Oral Cavity	8	16.7	2.4				
Parotid_L	8	11.5	10.0	16.6	9.4	0.003	AI‐Rad
Parotid_R^a^	8	9.9	6.4	12.1	7.1		
Prostate	5	5.6	2.1	8.6	4.8	0.120	
Rectum	10	13.0	8.9	30.2	14.8	0.017	AI‐Rad
SeminalVesicle	5	8.1	2.4	9.9	7.7	0.357	
SpinalCanal	10	4.9	0.9	113.2	13.0	0.052	
SpinalCord^a^	8	4.9	2.5	28.3	36.3		

**^a^** Data were not normally distributed, and therefore Student's *t*‐test was not performed.

Table 5 shows the sensitivity and precision of AI‐Rad and SS against the manual contours.

**TABLE 5:** Sensitivity and precision of AI‐Rad and SS against manual contours.

	AI‐Rad vs. Manual				SS vs. Manual			
	**Sensitivity**		**Precision**		**Sensitivity**		**Precision**	
**Structure**	**Mean**	**SD**	**Mean**	**SD**	**Mean**	**SD**	**Mean**	**SD**
A_Aorta	0.777	0.084	0.928	0.047				
Bladder	0.861	0.060	0.943	0.124	0.807	0.169	0.901	0.092
Brain	0.966	0.002	0.978	0.002	0.970	0.002	0.970	0.005
Brainstem	0.917	0.044	0.739	0.056	0.846	0.035	0.845	0.068
BreastL	0.915	0.038	0.869	0.071	0.941	0.044	0.764	0.088
BreastR	0.904	0.050	0.884	0.060	0.951	0.019	0.777	0.057
Esophagus	0.756	0.114	0.748	0.060				
Femur_L	0.896	0.050	0.978	0.009	0.891	0.133	0.911	0.041
Femur_R	0.912	0.038	0.969	0.022	0.887	0.134	0.915	0.039
Globe_L	0.849	0.071	0.946	0.039	0.843	0.072	0.764	0.070
Globe_R	0.830	0.076	0.962	0.035	0.801	0.090	0.719	0.058
Heart	0.887	0.030	0.981	0.006	0.920	0.037	0.870	0.060
Larynx	0.710	0.153	0.289	0.033				
Lens_L	0.640	0.123	0.863	0.150				
Lens_R	0.676	0.150	0.828	0.138				
Lung_L	0.985	0.009	0.932	0.043	0.951	0.033	0.984	0.016
Lung_R	0.986	0.014	0.946	0.033	0.946	0.035	0.984	0.011
Mandible	0.939	0.005	0.779	0.054				
OpticNrv_L	0.475	0.156	0.666	0.166	0.558	0.258	0.157	0.195
OpticNrv_R	0.363	0.145	0.752	0.091	0.533	0.193	0.207	0.158
Oral Cavity	0.847	0.051	0.805	0.049				
Parotid_L	0.638	0.080	0.895	0.114	0.747	0.097	0.675	0.070
Parotid_R	0.654	0.070	0.922	0.046	0.778	0.071	0.723	0.050
Prostate	0.779	0.122	0.925	0.052	0.691	0.230	0.890	0.161
Rectum	0.674	0.145	0.838	0.179	0.598	0.194	0.477	0.219
SeminalVesicle	0.887	0.135	0.547	0.168	0.734	0.235	0.537	0.274
SpinalCanal	0.976	0.042	0.539	0.076	0.768	0.076	0.599	0.041
SpinalCord	0.952	0.039	0.676	0.081	0.691	0.086	0.747	0.122
Average	0.809		0.826		0.803		0.734	

Table 6 shows the qualitative rating (Degrees of Editing) of each automated contour generated by AI‐Rad scored by the contouring RTT.

**TABLE 6:** Qualitative rating of automated contours generated by AI‐Rad.

Structure	No edits (*n*/%)	Minor edits (*n*/%)	Moderate edits (*n*/%)	Major edits (*n*/%)
A_Aorta	2 (40%)	3 (60%)	0	0
Bladder	4 (40%)	0	3 (30%)	3 (30%)
Brain	0	8 (100%)	0	0
Brainstem	1 (12.5%)	6 (75%)	1 (12.5%)	0
BreastL	5 (100%)	0	0	0
BreastR	5 (100%)	0	0	0
Esophagus	5 (100%)	0	0	0
Femur_L	10 (100%)	0	0	0
Femur_R	10 (100%)	0	0	0
Globe_L	8 (100%)	0	0	0
Globe_R	8 (100%)	0	0	0
Heart	3 (30%)	7 (70%)	0	0
Larynx	0	0	1 (12.5%)	7 (87.5%)
Lens_L	8 (100%)	0	0	0
Lens_R	8 (100%)	0	0	0
Lung_L	10 (100%)	0	0	0
Lung_R	10 (100%)	0	0	0
Mandible	0	0	8 (100%)	0
OpticNrv_L	7 (87.5%)	1 (12.5%)	0	0
OpticNrv_R	7 (87.5%)	1 (12.5%)	0	0
Oral Cavity	0	2 (25%)	6 (75%)	0
Parotid_L	6 (75%)	0	1 (12.5%)	1 (12.5%)
Parotid_R	6 (75%)	0	2 (25%)	0
Prostate	5 (100%)	0	0	0
Rectum	0	0	2 (20%)	8 (80%)
SeminalVesicle	3 (60%)	1 (20%)	1 (20%)	0
SpinalCanal	10 (100%)	0	0	0
SpinalCord	4 (50%)	4 (50%)	0	0

Table 7 compares the time required to review and modify the automated contours generated by AI‐Rad against the time required to manually delineate the same set of structures from scratch. The last two columns of the table list the *p*‐value of the pairwise comparison, and the superior method if the difference is statistically significant (*p* < 0.05).

**TABLE 7:** Comparison of AI‐Rad and manual contouring times.

	AI‐Rad time (seconds)		Manual time (seconds)			
Structure	Mean	SD	Mean	SD	*p*‐Value	Superior method Mean
A_Aorta	29	14	200	76	0.008	AI‐Rad
Bladder	66	52	67	30	0.924	
Brain	56	10	96	30	0.006	AI‐Rad
Brainstem	58	16	92	17	0.013	AI‐Rad
BreastL	23	3	228	42	0.000	AI‐Rad
BreastR	23	4	210	23	0.000	AI‐Rad
Esophagus	30	6	183	75	0.011	AI‐Rad
Femur_L	18	3	137	22	0.000	AI‐Rad
Femur_R	17	3	142	27	0.000	AI‐Rad
Globe_L	13	3	30	7	0.000	AI‐Rad
Globe_R	11	3	21	5	0.001	AI‐Rad
Heart	33	15	148	67	0.001	AI‐Rad
Larynx	125	37	69	10	0.007	Manual
Lens_L	9	2	27	12	0.007	AI‐Rad
Lens_R	11	3	23	8	0.005	AI‐Rad
Lung_L	24	4	85	57	0.008	AI‐Rad
Lung_R	24	5	123	53	0.000	AI‐Rad
Mandible	86	17	204	49	0.000	AI‐Rad
OpticNrv_L	17	3	37	8	0.000	AI‐Rad
OpticNrv_R	16	6	27	3	0.007	AI‐Rad
Oral Cavity	59	17	68	8	0.122	
Parotid_L	42	45	84	20	0.012	AI‐Rad
Parotid_R	36	32	72	17	0.002	AI‐Rad
Prostate	22	3	70	7	0.000	AI‐Rad
Rectum	102	36	76	17	0.085	
SeminalVesicle	26	9	55	17	0.077	
SpinalCanal	37	6	203	62	0.004	AI‐Rad
SpinalCord	48	13	116	13	0.000	AI‐Rad

**FIGURE 1:**
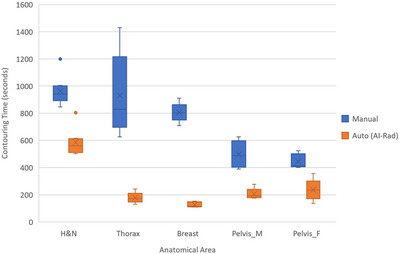
Comparison of automated and manual contouring times per anatomical area.

Figure 1 compares the average automated contouring time of AI‐Rad per anatomical area against that of manual contouring.

## 4 DISCUSSION

### 4.1 Quantitative and qualitative analysis of the quality of AI‐Rad automated contours

In this study, the quality of AI‐Rad for different anatomical areas was evaluated both quantitatively using DSC, precision, accuracy, and HD, and qualitatively using a subjective score (“Degree of Editing”) rated by the contouring RTT. Results are shown in Tables 3, 4, 5, 6.

DSC is widely utilized to assess the quality of contours. Although different studies have used a range of DSC thresholds,^33^, ^34^, ^35^ a value of 0.7 was selected in this case due to the inclusion of a large number of small volume structures, which can be over‐penalized by using a high DSC.^36^ In this study, of the 28 structures contoured by AI‐Rad, only five structures, namely Larynx (0.405), OpticNrv_L (0.531), OpticNrv_R (0.468), SeminalVesicle (0.652), and SpinalCanal (0.691), had a DSC lower than 0.7. Alternatively, from the results of the qualitative analysis, structures that required moderate or major editing in more than 50% of the instances included Bladder (60.0%), Larynx (100.0%), Mandible (100.0%), Oral Cavity (75.0%), and Rectum (100.0%).

**FIGURE 2:**
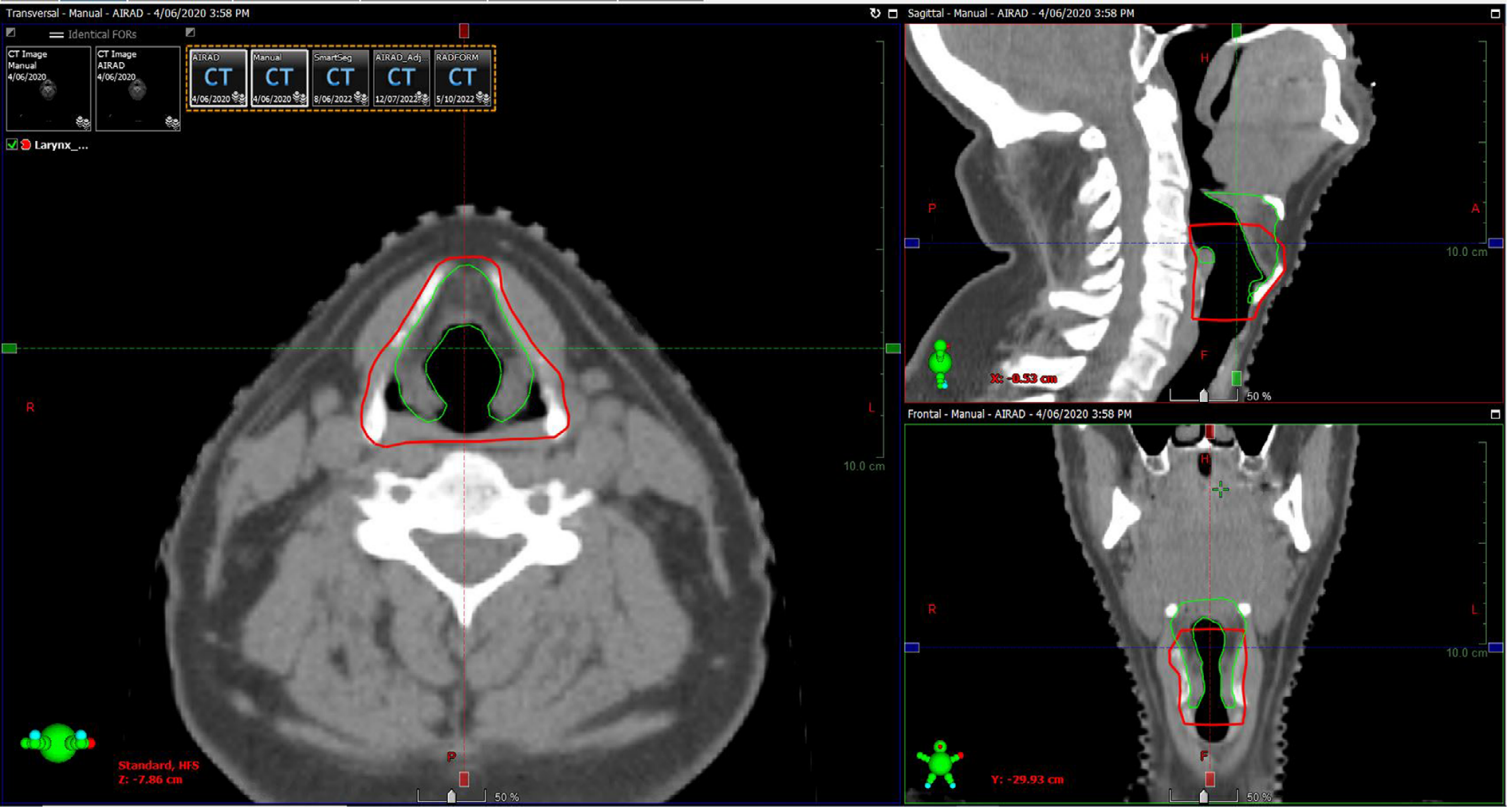
Comparison of the larynx contour generated by AI‐Rad (Green) and that delineated manually (Red). Disagreement between the two contours is mainly contributed by different definitions of the boundaries of the structure.

The only structure that had both a low DSC and a low qualitative rating was Larynx. Similar findings have been reported by the literature, suggesting that the lack of standardization on larynx boundaries and the complexity of the small structure makes its automated segmentation on CT difficult.^38^ Similarly, a closer review of the results in this study indicated that the low agreement between the larynx contour generated by AI‐Rad and that delineated manually was predominantly caused by different definitions of the boundaries of the structure. Within the author's department, larynx is defined to start with epiglottis as the most superior point and include the thyroid cartilage whilst excluding the hyoid bone and the laryngopharynx, whereas the posterior and inferior edges are defined by the pharyngeal constrictor and the cricoid cartilage. However, the Larynx structure contoured by AI‐Rad starts more superiorly and excludes both the thyroid cartilage and the airway, thereby the low DSC. An example is shown in Figure 2.

Figure 2 Comparison of the larynx contour generated by AI‐Rad (Green) and that delineated manually (Red). Disagreement between the two contours is contributed mainly by different definitions of the boundaries of the structure.

Among the other structures with a low DSC, the left and right optic nerves and the spinal canal never required any manual editing, and only one case of the seminal vesicle required manual editing as per the qualitative score. The inconsistency between the DSC and the qualitative score was predominantly caused by differences in the length of the contour in the superior‐inferior direction, with manual contours being consistently shorter than automated ones. This is because the RTT only contoured these structures on slices where the target structures were located, whereas the AI‐Rad tool contoured all the slices where the structures were present. To further validate the observation, the above structures generated by AI‐Rad were cropped to the same length of the corresponding manual contours, followed by re‐performing the DSC analysis. For the above structures, the DSC significantly improved after cropping (e.g., for Spinal Canal, DSC increased from 0.691 to 0.854; *p* = 0.012 < 0.05).

Similar under‐segmentation has been reported by a previous study.^32^ The fact that manual contours were under‐delineated in the superior‐inferior direction was caused by time constraints RTTs and ROs frequently encounter during clinical workflows. This can affect the accuracy of the DVH of a structure and exhibit a significant impact on particularly small serial structures such as optic nerves. Therefore, there is a need to utilize qualitative metrics in addition to quantitative metrics such as DSC,^39^ which can exaggerate the difference between manual and automated contours if not interpreted carefully. The observation also highlights the benefit of introducing automated contouring solutions, allowing users to amend on comprehensively delineated structures, thereby reducing the risk of time‐based contouring errors while maintaining efficiency.

Contours for the bladder, mandible, oral cavity, and rectum all required significant editing when qualitatively evaluated despite acceptable DSCs. The commonality between these organ types is the large structure volume, further highlighting the insensitivity of the DSC metric to inaccuracies in large structures. Poor contouring accuracy for the oral cavity and rectum could be attributed to low‐contrast tissue boundaries, which previous studies have linked to worse AI contouring performance.^32^, ^33^, ^38^ Poor results for the bladder and mandible may suggest necessary improvements to the structure models, with alternative AI models demonstrating greater accuracy when generating contours across patient cohorts than demonstrated in this work.^40^, ^41^ For AI‐Rad, improvements of the structure models can be requested but are not adjustable by the user. The inability for individual sites to improve specific structure models highlights a limitation of this implementation model.

### 4.2 Comparison between AI‐Rad and SS

Of the DSCs of the 21 contours that both AI‐Rad and SS could generate, the data of 18 structures passed the normality test, warranting the use of Student's *t*‐test for pairwise comparison. Among them, 10 showed significant differences, all favoring AI‐Rad, which indicated its superior performance over SS.

The HD data were less normally distributed, with only 16 structures passing the normality test. Among these 16 structures, nine showed significant differences, with three favoring SS and six favoring AI‐Rad. However, for the three structures that favored SS, qualitative scores in Table 6 showed that only one case of Brainstem required moderate editing, whereas neither Lung_L nor Lung_R required manual editing in any cases. This observation indicated that despite the statistically inferior HD values of AI‐Rad in these structures, the actual clinical impact was small.

Previous studies have indicated that atlas‐based automated contouring software performed poorly in structures with a low‐contrast tissue boundary that was hardly distinguishable from surrounding tissues, especially in the pelvic area.^32^, ^33^, ^38^ This study found that, compared to SS, AI‐Rad performed substantially better in contouring such structures including the bladder and the rectum, although manual editing was still required for the automated contours to be clinically accepted.

Comparison of the precision and the sensitivity of the two automated contouring tools suggested that whilst the sensitivity of AI‐Rad and SS was similar (0.809 vs. 0.803), the precision of AI‐Rad was higher (0.826 vs. 0.734, *p* < 0.005), suggesting a tendency of SS to over‐contour compared to AI‐Rad.

The clinical implementation of SS requires training the atlas with user‐defined datasets, whereas the ML database of AI‐Rad is pre‐configured by the vendor and therefore requires no user input. Additionally, AI‐Rad does not require any user interactions other than uploading the CT dataset, whereas SS requires several steps of user interactions before generating results. Therefore, it is expected that the clinical implementation of AI‐Rad is easier than that of SS.

### 4.3 Timing analysis

Among the 28 structures, 24 (85.7%) showed differences between the automated and the manual contouring times, with 23 (82.1%) favoring AI‐Rad and only 1 (4.2%, Larynx) favoring manual contouring. Similar observations have been reported by the literature, that novel AI contouring tools are more efficient in contouring various organs, thereby allowing the comprehensive delineation of multiple structures that facilitates not only planning, but also reporting in radiotherapy.^9^, ^32^, ^33^ In addition, Figure 1 shows that for all five anatomical areas, the time spent by AI‐Rad is consistently shorter than that by manual segmentation, with more significant time savings observed in the Thorax and the Breast areas. The average time saving for each area, calculated from the difference between the mean times of the two methods in contouring all structures for a specific anatomical area, is 379s for H&N, 753s for Thorax, 679s for Breast, 291s for Pelvis_M, and 210s for Pelvis_F patients, respectively.

### 4.4 Study limitations

One limitation of the study was that the contours of a single RTT were adopted as the ground truth to evaluate the quality of automated contours, which may introduce bias to the results. Therefore, although this study found that the quality of automated contours generated by AI‐Rad was better than that of those generated by SS, further investigation that utilizes ground truth datasets defined by the common agreement of multiple users may be required. In addition, the performance of AI‐Rad in different structures, patient cohorts, and imaging modalities such as cone‐beam CT (CBCT) should be performed with a larger sample size.

## 5 CONCLUSION

This study evaluated the performance of AI‐Rad, a ML‐based AI contouring tool, both quantitatively and qualitatively. The results suggested that most of the automated contours generated by AI‐Rad were clinically acceptable and required minimal manual editing. In addition, AI‐Rad significantly outperformed SS, an atlas based commercial AI contouring software, in over half of the compared contours. Timing analysis favored AI‐Rad over manual contouring, with the former achieving significant time saving in several structures, especially in the Thorax area. In summary, AI‐Rad is a promising automated contouring solution that can generate clinically acceptable contours and achieve time savings. To the authors’ knowledge, this is the first contour‐comparison study that has explored the performance of AI‐Rad, a novel AI contouring tool, and investigated possible time savings it may achieve in the clinical environment.

## AUTHOR CONTRIBUTIONS

Yunfei Hu, Huong Nguyen, Claire Smith, Tom Chen, and Trent Aland conceived of the presented idea. Yunfei Hu, Huong Nguyen, Claire Smith, Tom Chen, and Trent Aland carried out the experiment. Yunfei Hu, Mikel Byrne, and Ben Archibald‐Heeren performed related calculations and statistical analyses. James Rijken developed the C# application for data collection. Yunfei Hu wrote the manuscript with support from Mikel Byrne, Ben Archibald‐Heeren, and James Rijken. Trent Aland supervised the project.

## CONFLICT OF INTEREST STATEMENT

The authors declare that there is no duality of interest that they should disclose.

## Data Availability

The data that support the findings of this study are available from the corresponding author upon reasonable request.
